# Condition-Dependent Cell Volume and Concentration of *Escherichia coli* to Facilitate Data Conversion for Systems Biology Modeling

**DOI:** 10.1371/journal.pone.0023126

**Published:** 2011-07-29

**Authors:** Benjamin Volkmer, Matthias Heinemann

**Affiliations:** 1 Institute of Molecular Systems Biology, ETH Zurich, Zurich, Switzerland; 2 Molecular Systems Biology, Groningen Biomolecular Sciences and Biotechnology Institute, University of Groningen, Groningen, The Netherlands; 3 Life Science Zurich PhD Program on Systems Biology of Complex Diseases, Zurich, Switzerland; German Cancer Research Center, Germany

## Abstract

Systems biology modeling typically requires quantitative experimental data such as intracellular concentrations or copy numbers per cell. In order to convert population-averaging omics measurement data to intracellular concentrations or cellular copy numbers, the total cell volume and number of cells in a sample need to be known. Unfortunately, even for the often studied model bacterium *Escherichia coli* this information is hardly available and furthermore, certain measures (e.g. cell volume) are also dependent on the growth condition. In this work, we have determined these basic data for *E. coli* cells when grown in 22 different conditions so that respective data conversions can be done correctly. First, we determine growth-rate dependent cell volumes. Second, we show that in a 1 ml *E. coli* sample at an optical density (600 nm) of 1 the total cell volume is around 3.6 µl for all conditions tested. Third, we demonstrate that the cell number in a sample can be determined on the basis of the sample's optical density and the cells' growth rate. The data presented will allow for conversion of *E. coli* measurement data normalized to optical density into volumetric cellular concentrations and copy numbers per cell - two important parameters for systems biology model development.

## Introduction

Systems biology ultimately tries to attain quantitative understanding about biological systems [1]. For this endeavor, mathematical models are important tools. For their development, most often quantitative data on intracellular concentrations or copy numbers of proteins, metabolites or other biomolecules are needed (e.g. as in [2], [3], [4]). Current omics technologies resemble a great source for such data [5], [6], [7]. However, these measurement techniques typically only sample at the cell population-level, thus yielding molecule copy numbers (moles) per sample (i.e. per cell dry weight or per optical density), while for mathematical modeling intracellular molecule concentrations or absolute intracellular molecule copy numbers are needed. In order to convert the current omics data into such units, knowledge of the volume and number of the sampled cells is instrumental. This information is however lacking even for the well-studied model organism *E. coli*.

Cell dimensions and cell volume have often been determined by coulter counter measurements [8] or by electron microscopy, where the cells undergo extensive preparation procedures before they can be observed, often introducing a measurement bias [9]. For *E. coli*, cell volume has also been determined by measurement of the volume of a cell pellet and subsequent division of the volume by the cell number [10]. Microscopy as another measurement option requires cells to be held in place and high magnification [11], [12] and is rather laborious. Using these different methods, the average length of the rod-shaped bacterium *E. coli* was determined to lie between 1.6 and 3.1 µm [12], [13], the average width was determined as 0.7–1.1 µm [11], [14] and the volume was determined to range from 0.5–4 µm^3^ [10], [15], [16], [17]. The differences between the determined cell lengths and volumes can be explained by the increase in cell length and therefore volume with growth rate [18]. Unfortunately, information about the cell volume is only available for a limited number of growth conditions.

To infer intracellular concentrations and molecule numbers from population-level measurements, the total cell volume and total number of cells in the sample need to be known, respectively. The total cell number in a sample is dependent on the bacterial cell density. Bacterial density is typically measured on the basis of determining the amount of transmitted or scattered light. Such optical density (OD) measurements do not measure the number of cells directly but correlate the absorption of light to the cell concentration. In preliminary experiments, we observed that the OD-specific concentration of *E. coli* cells in a culture (i.e. the number of cells per milliliter at an OD of 1 measured at 600 nm) varies when the cells are grown in different conditions. Therefore, the number of cells in a sample cannot simply be determined by measuring the OD of the culture. Unfortunately today, there is no data available that describes the dependence between the number of cells and the OD when cells grow in different conditions.

In order to make omics data generated for *E. coli* accessible to modeling endeavors, in this work we determined the optical density, cell concentration and cell size of *E. coli* BW25113, a commonly used K-12 strain in several systems biology programs [7], [19], [20], when grown under 22 different growth conditions. We report the growth-condition dependent cell dimensions and show that the OD-specific cell concentration decreases with increasing growth rate. Further, we show that OD correlates with the total cell volume in a sample. We derive an empirical equation that can be used to calculate both the cell concentration in a sample and the total cell volume from the OD value and the cells' growth rate. Comparison experiments using the MG1655 strain show that these results are generally valid for *E. coli*. Altogether, the presented results now allow for correct determination of cellular concentrations or copy numbers from typical omics data.

## Results

To determine condition-specific cell volume and concentration data, we selected those conditions that are most commonly used in the literature for experimental data acquisition in *E. coli* systems biology endeavors. We grew the cells in steady-state on complex medium (LB), on M9 minimal medium containing different carbon sources with various entry points into metabolism and on M9 minimal with different carbon sources with amino acids added to be able to sample a larger range of growth rates. Furthermore, the cells grown in glucose minimal medium were also exposed to different stress conditions (pH, temperature, osmotic and oxygen stress) and also subjected to four different growth rates in a chemostat. Lastly, we also analyzed cells that had entered stationary phase. The OD values at 600 nm of the cultures were determined by spectrophotometry, the OD-specific cell concentration was analyzed by flow cytometry and the cell volume by fluorescence microscopy. Besides the *E. coli* K-12 strain BW25113, the strain MG1655 was analyzed for a selected subset of conditions to test whether the results obtained with BW25113 are transferable also to other *E. coli* strains.

### Cell size and volume

The average cell size was determined from microscopic images of cells taken directly from steady state cultures. To facilitate image analysis we used fluorescence microscopy and cells expressing GFP from a plasmid under the control of the *pykF* promoter, allowing precise software-based measurement of both the long and short axis of the cells. The cell volume was calculated by approximating the cell shape as a cylinder capped by two half-spheres as done previously [9], [12], [17].

Consistent with the literature, the length of the cells varied with the condition, between 1.6 µm for stationary cells and 3.9 µm for cells growing on LB medium (Table 1). Since *E. coli* cells grow by elongation, the cell length of individuals varies greatly in a population, thereby leading to a large standard deviation of the average cell length. Also in agreement with the literature, we found the cell width to be condition-independent (1.26 µm ±0.16 µm). This value is slightly higher than what was previously reported. However, a control experiment using fluorescent beads of known size confirmed the correctness of our size measurements. Presumably, earlier reported smaller widths might have been caused by the fact that fixation by formaldehyde for electron microscopy leads to cell shrinkage and reduces the measured volume [9], [16]. To determine the cell volume, we used an average cell width of 1.26 µm for all conditions, obtaining a volume range from 1.5 to 4.4 fl (1 fl  = 1 µm^3^).

**Table 1:** Measured cell parameters on different growth conditions.

	growth condition	growth rate [h^−1^]	cell length [µm]	cell width [µm]	single cell volume [fl]	OD-specific cell concentration [10^8^cells·ml^−1^·OD^−1^]	OD-specific total cell volume [µl·ml^−1^·OD^−1^]
complex medium	LB	1.61±0.05	3.9±0.9	1.3±0.2	4.4±1.1	7.8±0.8	3.4
	LB *MG1655*	1.62±0.04	3.5±0.9	1.4±0.1	3.9±1.2	7.5±0.8	2.9
	glucose+AA	1.49±0.05	3.5±1.0	1.5±0.1	4.0±1.3	5.9±0.6	2.4
	mannose+AA	1.28±0.07	3.7±0.9	1.5±0.2	4.1±1.2	6.3±0.6	2.5
	glycerol+AA	1.26±0.04	3.5±0.9	1.5±0.1	3.9±1.2	8.2±0.8	3.2
carbon sources	acetate	0.29±0.02	2.3±0.6	1.2±0.1	2.4±1.3	16.8±1.7	4.0
	fumarate	0.47±0.03	2.4±0.6	1.1±0.1	2.4±1.2	17.0±1.7	4.1
	galactose	0.17±0.02	2.0±0.5	1.1±0.1	1.9±1.2	19.9±2.0	3.8
	glucose	0.60±0.05	3.0±0.7	1.4±0.2	3.2±1.2	11.1±1.1	3.6
	glucose *MG1655*	0.67±0.05	2.8±0.7	1.4±0.2	3.0±1.3	11.0±1.1	3.3
	glucosamine	0.39±0.03	2.7±0.7	1.3±0.1	2.9±1.3	12.2±1.2	3.5
	glycerol	0.47±0.03	2.3±0.6	1.2±0.1	2.3±1.3	19.6±2.0	4.5
	pyruvate	0.40±0.03	2.2±0.6	1.0±0.1	2.1±1.2	21.0±2.1	4.5
	succinate	0.49±0.02	2.4±0.6	1.1±0.2	2.4±1.3	16.7±1.7	4.1
stress conditions on glucose	anaerobic	0.55±0.01	2.8±0.7	1.3±0.2	2.9±1.2	10.4±1.0	3.1
	50 mM NaCl	0.65±0.02	2.6±0.7	1.3±0.2	2.8±1.2	11.3±1.1	3.1
	pH 6	0.50±0.11	2.9±0.8	1.3±0.2	3.1±1.3	10.5±1.1	3.3
	42°C	0.65±0.02	2.7±0.7	1.3±0.2	2.8±1.2	11.0±1.1	3.1
fixed growth rate on glucose	chemostat µ = 0.5	0.50	2.5±1.2	1.2±0.2	2.6±1.9	13.4±1.3	3.5
	chemostat µ = 0.35	0.35	2.4±1.0	1.0±0.1	2.4±1.7	19.7±2.0	4.8
	chemostat µ = 0.20	0.20	2.2±1.0	1.0±0.1	2.2±1.8	20.6±2.1	4.5
	chemostat µ = 0.12	0.12	2.1±1.1	1.1±0.1	2.1±1.9	23.0±2.3	4.9
starved cells	stationary 1 day	0.00	1.6±0.4	1.1±0.2	1.5±1.2	21.9±2.2	3.3
	stationary 3 days	0.00	1.7±0.3	1.4±0.1	1.6±1.1	22.9±2.3	3.7

Unless indicated otherwise the data is for the *E. coli* strain BW25113. Errors are given as standard deviations.

**Figure 1:**
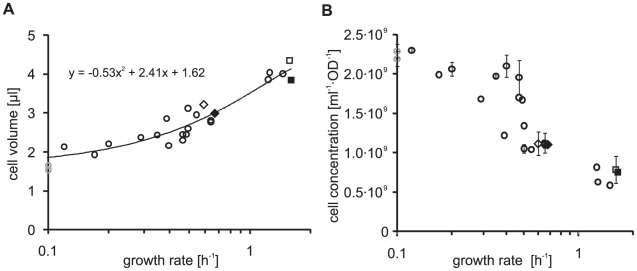
Cell volume and OD-specific cell concentration of cells grown in 22 different conditions. Diamonds: BW25113 (empty) and MG1655 (filled) grown on glucose minimal medium. Squares: BW25113 (empty) and MG1655 (filled) grown on LB medium. Grey circles: Non-growing stationary cells plotted at a growth rate of 0.1 on the logarithmic x-axis. A: Cell volume plotted against the growth rate. Dashed line: polynomial fit. B: OD-specific cell concentration (cells/[ml⋅OD]) plotted against the growth rate. Error bars indicate the standard deviation of at least two independent experiments (only available for a subset of conditions). The day-to-day variability in the cell concentration measurements was found to be less than 10% (data not shown).

As can be seen in Figure 1A, there is a clear growth rate dependence of the cell volume; cells with a higher growth rate also have a larger volume. This trend is visible across all conditions, and thus knowing the growth rate is sufficient to the estimate an individual cell's volume (e.g. with equation in Figure 1A).

### OD-specific cell concentration decreases with growth rate

In order to convert metabolite or protein data measured per OD unit (as for example reported in [21], [22], [23]) into intracellular molar concentrations, often a fixed conversion is assumed for different growth conditions [7], [24]. In doing so, the changes in the cells' sizes and the OD-specific cell concentration associated with these different conditions are disregarded.

In order to enable the calculation of intracellular molar concentrations in a condition-dependent manner, we determined the OD-specific cell concentration by flow cytometry for different conditions and report this data as cell concentrations normalized to the corresponding OD values (cells·ml^−1^·OD^−1^) to be able to compare the different conditions with each other.

As can be seen in Figure 1B the cell concentration per OD decreases with increasing growth rate. Between cells with the lowest and the highest growth rate the OD-specific cell concentration changes by a factor of four, demonstrating that the number of cells in a sample varies although the OD is identical. Therefore, when determining intracellular molecule concentrations or copy numbers, either the cells need to be counted directly or the OD and the number of cells per OD need to be known.

### Total cell volume in a sample correlates with culture OD

**Figure 2:**
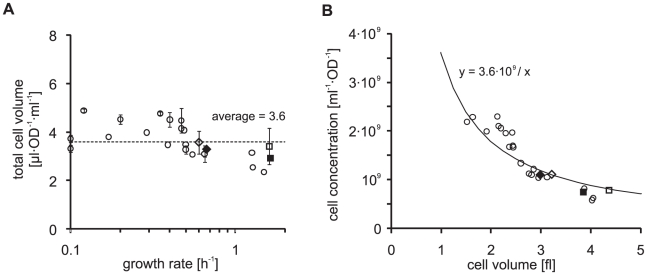
The OD-specific total cell volume is condition independent. Diamonds: BW25113 (empty) and MG1655 (filled) grown on glucose minimal medium. Squares: BW25113 (empty) and MG1655 (filled) grown on LB medium. A: Total cell volume per ml⋅OD is plotted against the growth rate. Grey circles: Non-growing stationary cells plotted at a growth rate of 0.1 on the logarithmic x-axis. Error bars indicate the standard deviation introduced by the variation in the OD-specific cell concentration measurement (only available for a subset of conditions). B: Cell concentration (cells/[ml⋅OD]) is plotted against cell volume. Continuous line: fixed volume of 3.6 µl divided by the condition dependent cell volume.

Now that we know that both the cell volume and OD-specific cell concentration are growth rate dependent, we asked whether knowing the OD may be sufficient to allow the determination of the total cell volume in a sample – a correlation that one could use to convert any OD-specific omics measurement into actual molar concentrations. When multiplying the cell volume (Figure 1A) and the OD-specific cell concentration (cells·ml^−1^·OD^−1^; Figure 1B) one can obtain an OD-dependent total volume of the cells (µl·ml^−1^·OD^−1^; Figure 2A).

It turns out that independent of the condition or growth rate we obtain a almost constant number for this value (with the highest and lowest values differing only by a factor of two, which can be considered marginal given the higher condition-dependent variation in cell volumes and OD-specific cell concentrations). This means that the total cell volume per OD is basically constant for a wide range of different cultivation conditions and that OD measurements can in fact be used to estimate the total cell volume in a sample. With the spectrophotometer used in this study, one milliliter of culture at OD 1 would correspond to a total cell volume of approximately 3.6 µl. This value can now be used to estimate volumetric concentrations of cellular molecules. With the equation shown in Figure 2B it is further possible to infer the OD-specific cell number in a sample, thereby allowing the determination of cellular copy numbers from OD-normalized data.

### Cell volume at different growth conditions can be determined by flow cytometry

Now that we have determined the condition-specific cell volumes (by microscopy), we asked whether we could determine these volumes also by flow cytometric measurements. For bacterial cells, a relationship between bacterial volume and forward scatter (FSC) has been observed [25], [26], [27]. For mammalian cells, FSC is used to measure cell size, while the sideward scatter (SSC) is used to measure granularity of the cells [28].

**Figure 3:**
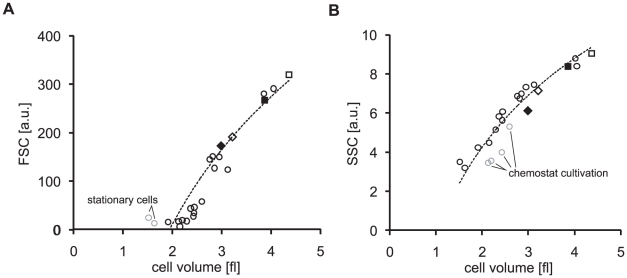
Forward and sideward scatter correlate with cell volume. Diamonds: BW25113 (empty) and MG1655 (filled) grown on glucose minimal medium. Squares: BW25113 (empty) and MG1655 (filled) grown on LB medium. FSC (A) and SSC (B) values plotted against single cell volumes. Grey circles: stationary cells.

When plotting the FSC and SSC values measured at different conditions against the cell volumes, we find that both scatter measurements – within limits - correlate with cell volume (Figure 3). Exceptions for the FSC measurements are the stationary cells, since the cell volume of the stationary cells is very low and many of the cells are just around the detection limit of the instrument (Figure 3A). Thus, cells below a certain volume may escape detection and in turn result in a too large FSC value. The correlation of the SSC-values with cell volume (Figure 3B) show that the chemostat cultivation condition, where the growth rate is controlled by nutrient limitation, may lead to an increase of granularity and thereby result in an slightly altered SSC signal. The data shown in Figure 3 nevertheless indicates that the volume of *E. coli* cells can be estimated – within limits - by both FSC and SSC measurements regardless of the growth condition. However, it has to be noted that the scatter signal needs to be calibrated for volume measurements as these readings are dependent on flow cytometer settings. For doing this calibration the data presented in Figure 1A or Figure 2B can be used.

### Conclusions

Systems biology modeling endeavors typically require experimental data on either molecule copy numbers per cell or volumetric concentrations e.g. in terms of mol/cell/volume. Unfortunately, omics data are often only referenced to OD values making it difficult or impossible for the modeler to directly use the data. Here, we addressed this problem and shed light on the dependencies between OD, growth rate, cell number and cell size of *E. coli* for a large number of experimental conditions. Our results provide the means to covert population data into a per cell format and at the same time to correct for different cell sizes and OD-specific cell concentrations at different conditions such that now frequently reported experimental omics data can be converted into units that can be used for systems biology modeling.

## Materials and Methods

### Strains and plasmids

The wild type (wt) *Escherichia coli* K-12 strain BW25113 harboring a reporter plasmid for *pykF*, which contained the *E. coli pykF*-promoter region followed by a *gfp* gene [29] was used throughout this work. Where indicated, the wild-type strain MG1655 (MG) was used for comparison; equally containing the *pykF* reporter plasmid.

### Media and cultivation

All water for media components was purified (Nanopure type I ultrapure water system, Barnstead) and autoclaved. Salt solutions are always aqueous solutions if not indicated otherwise. LB-medium was prepared as follows: Five grams of yeast extract (BD), 10 g Tryptone (BD) and 10 g NaCl were dissolved in one liter of purified water and the mixture sterilized by autoclaving. LB-plates were produced by adding 20 g agar (BD) to the LB-medium mixture before autoclaving. Before pouring the plates Kanamycin (25 µg/ml) was added after the mixture had cooled to approximately 50°C. M9 minimal medium without carbon source was prepared in the following way: To 700 ml of autoclaved, purified water, 200 ml of 5x base salt solution (211 mM Na_2_HPO_4_, 110 mM KH_2_PO_4_, 42.8 mM NaCl, 56.7 mM (NH_4_)_2_SO_4_, autoclaved), 10 ml of trace elements (0.63 mM ZnSO_4_, 0.7 mM CuCl_2_, 0.71 mM MnSO_4_, 0.76 mM CoCl_2_, autoclaved), 1 ml 0.1 M CaCl_2_ solution (autoclaved), 1 ml 1 M MgSO_4_ solution (autoclaved), 2 ml of 500x thiamine solution (1.4 mM, filter sterilized) and 0.6 ml 0.1 M FeCl_3_ solution (filter sterilized) were added. The resulting solution was filled up to 1 liter with water. All chemicals used were obtained from Sigma-Aldrich.

In order to prepare M9 minimal medium with a specific amount of carbon source, aqueous stock solutions were used. Aqueous stock solutions were prepared for every carbon source, adjusted to pH 7 by titration with 1 M sodium hydroxide or fuming hydrochloric acid. M9 minimal medium was complemented with carbon source by mixing appropriate amounts of carbon source free M9 minimal medium and carbon source stock solutions. The medium was always filtrated prior to use (Steritop-GP 500 ml, Millipore). The following carbon sources and concentrations were used: acetate (sodium acetate, 3.5 g/L), fumarate (disodium fumarate, 2.8 g/L), galactose (2.3 g/L), glucose (5 g/L), glucosamine (2.1 g/L), glycerol (2.2 g/L), pyruvate (sodium pyruvate, 3.3 g/L), succinate (disodium succinate hexahydrate, 5.7 g/L). For chemostat growth only 1 g/L of glucose was used. Medium for the cells grown with osmotic stress was supplemented with NaCl to a concentration of 50 mM; for the cells grown with pH stress, fuming hydrochloric acid was titrated to the medium until a pH of 6 was reached. When appropriate, a stock solution containing all amino acids (AA) was added to the M9 minimal media containing either glucose, mannose or glycerol. The AA concentrations in these media are indicated in Zaslaver et al. [30].

Cells were grown as follows. Cells were reconstituted from −80°C stocks using LB-agar plates with Kanamycin added, grown on the plate overnight and kept at 4°C for a maximum of three weeks. Preculture: a single colony was picked from a plate and grown overnight in 5 ml M9 glucose medium in a 14 ml preculture tube with a loosely closed cap (Greiner bio-one) at 37°C, 300 rpm and 5 cm shaking diameter (ISF-4-V shaker, Kühner). Batch cultures: Cells from a preculture were re-inoculated into 50 ml of pre-warmed medium in a 500 ml unbaffled wide-neck Erlenmeyer flask covered by a 38 mm silicone sponge closure (BellCo glass) and grown at 37°C, orbital shaking at 300 rpm and 5 cm shaking diameter (ISF-4-V, Kühner). To ensure steady state-growth, the cells were first grown over-night and passaged into a second shake-flask containing fresh medium the next day thus having undergone at least 10 divisions when measured. Cells undergoing temperature stress were grown at 42°C. Anaerobic cultures were grown in 200 ml closed bottles, after residual oxygen was removed by flushing the medium with nitrogen for 30 minutes. Cells grown in a mini-chemostat as described in Nanchen et al. [31] were inoculated from a preculture to an OD of 0.1 and allowed to grow in batch mode to an OD of around 0.8 before dilution (rates: 0.12, 0.2, 0.35, 0.5) was started. Stationary phase cells were continuously shaken after reaching stationary phase for either 1 or 3 days. Additionally cells of all conditions except for the stress conditions, chemostat growth and growth of the MG1655 strain, were cultivated using an automated cultivation device (Tecan Infinite 200 Pro plate reader). For this, cells from the second shake flask culture were washed twice by centrifuging of 1 mL of the culture and resuspending the pellet in 1 mL M9 minimal medium without carbon source. From the washed cells 4 µL of culture were inoculated into a well on a 96-well plate (Nunc) with 196 µL of medium. The plate was covered with its transparent plastic cover and sealed with parafilm. Cultivation was done at the maximal linear shaking speed (160 min^−1^, 1 mm displacement). The cells were grown to stationary phase or for at least 50 hours to ensure observation of steady state growth.

### Measurement of growth rate

For all shake flask batch cultures the OD was determined using a spectrophotometer at 600 nm (Pharmacia biotech Novaspec II). Samples were diluted with minimal medium to an OD value below 0.2. The growth rate of the cultures was determined from samples taken over time at OD-values from 0.05 to 0.75. The growth rates were additionally determined for selected conditions using the plate reader with the following settings for OD measurements (Interval time 5 min, shaking 4:42, reading (no shaking): 18 s; number of flashes 1; wavelength 600 nm, bandwidth 9 nm). The measured OD-values were corrected for the non-linearity of the device using an empirical function derived from samples with known OD-values (measured by spectrometry) from 10 to 0.001. The growth rates obtained by the plate reader were in excellent accordance with the values obtains for shake flask cultivation and are the ones reported in this work.

### Cell volume determination by microscopy

At an optical density of around 0.5, cells were harvested for cell volume determination. Cells were analyzed by fluorescence microscopy 1 to 3 minutes after removal from the shake flask without putting the cells on ice in order to minimize possible changes in cell volume induced by storage of the cells, e.g. by temperature change. 3 µl of the bacterial culture was spotted onto a coverglass and then covered with a thin agarose pad (2% agarose in M9 minimal medium without carbon source) to prevent cell movement. Brightfield DIC and green fluorescence images with a resolution of 0.092 µm/pixel were recorded with a Nikon Eclipse T*i*-E microscope (objective: CFI planapochromat 100x VC oil, camera: Hamamatsu ORCA C4742-95-12ER).

Fluorescence images were sharpened using an unsharp mask filter and brightness and contrast were enhanced (Photoshop, Adobe Systems). Adjacent cells were manually separated by drawing a line between the neighboring cells. For each condition, the dimensions of at least 200 individual cells were measured. To test the accuracy of the microscope size measurements, we also recorded images of fluorescent spherical beads (absolute counting beads, Countbright, Invitrogen) with a diameter of 7 µm. The cells and beads were automatically identified and their length and width was determined using Cellprofiler [32]. The determined bead size was 7.0±1.8 µm from a sample number of 15 beads. Calculation of the cell volume (V) from the length (l) and width (w) of the cells was done by assuming the cells to have the shape of a cylinder capped by two half-spheres and the resulting formula: V  =  π·w^2^·(l-w/3)/4 [9], [12], [17].

### Cell counting by flow cytometry

For cell concentration determination, cells were harvested at an optical density of around 0.5 and stored on ice to stop further cell division. For analysis, samples were diluted with carbon source free M9 minimal medium to an OD value of around 0.001, corresponding to a cell density of approximately 10^6^ cells/ml. Prior to measurement, 20 µl of gently vortexed counting beads (Countbright, Invitrogen) were added to 380 µl of cell suspension. Samples were vortexed for at least 5 seconds and then immediately measured for 1 minute without gating. A FACS Calibur flow cytometer (BD Biosciences) was used in this work. The instrument settings were the following: Flow rate: high, FSC: E02, SSC: 327, FL-1: 999, FL-2: 700: all log scale. Primary parameter: SSC, threshold: 50. For every condition, approximately 30′000 cells were counted.

Analysis of the data was done with FlowJo (Version 8.2, Tree Star) and determination of the absolute cell concentration in a sample was performed in the following way: (i) the total number of cells counted in a sample was determined by manual gating of the cells in a FL-1 over SSC dotplot, (ii) the total number of beads counted in the sample was determined by manual gating of the beads in a FSC over SSC dotplot, (iii) the measured volume was calculated from the total number of beads measured, and (iv) division of the measured cell number by the measured volume yielded the absolute concentration of cells in a sample. The day-to-day variability in the cell concentration measurements was found to be not more than 10%.
